# Evidence of a causal relationship between high serum adiponectin levels and increased cardiovascular mortality rate in patients with type 2 diabetes

**DOI:** 10.1186/s12933-016-0339-z

**Published:** 2016-01-27

**Authors:** Lorena Ortega Moreno, Massimiliano Copetti, Andrea Fontana, Concetta De Bonis, Lucia Salvemini, Vincenzo Trischitta, Claudia Menzaghi

**Affiliations:** Research Unit of Diabetes and Endocrine Diseases, IRCCS Casa Sollievo della Sofferenza, Viale Padre Pio, 71013 San Giovanni Rotondo, Italy; Unit of Biostatistics, IRCCS Casa Sollievo della Sofferenza, San Giovanni Rotondo, Italy; Department of Experimental Medicine, Sapienza University of Rome, Rome, Italy

**Keywords:** Adiponectin, ADIPOQ, Mortality, Mendelian randomization, Type 2 diabetes

## Abstract

**Background:**

Despite its beneficial role on insulin resistance and atherosclerosis, adiponectin has been repeatedly reported as an independent positive predictor of cardiovascular mortality.

**Methods:**

A Mendelian randomization approach was used, in order to evaluate whether such counterintuitive association recognizes a cause-effect relationship. To this purpose, single nucleotide polymorphism rs822354 in the *ADIPOQ* locus which has been previously associated with serum adiponectin at genome-wide level, was used as an instrument variable. Our investigation was carried out in the Gargano Heart Study-prospective design, comprising 356 patients with type 2 diabetes, in whom both total and high molecular weight (HMW) adiponectin were measured and cardiovascular mortality was recorded (mean follow-up = 5.4 ± 2.5 years; 58 events/1922 person-year).

**Results:**

The A allele of rs822354 was associated with both total and HMW adiponectin [β (SE) = 0.10 (0.042), p = 0.014 and 0.17 (0.06), p = 0.003; respectively]. In a Poisson model comprising age, sex, smoking habits, BMI, HbA1c, total cholesterol, HDL-cholesterol, triglycerides, insulin therapy and hypertension, both rs822354 (IRR = 1.94, 95 % CI 1.23–3.07; p = 0.005), as well as the genetic equivalent of total adiponectin change (IRR = 1.07, 95 % CI 1.02–1.12; p = 0.003) were significantly associated with cardiovascular mortality. The observed genetic effect was significantly greater than that exerted by the genetic equivalent change of serum adiponectin (p for IRR heterogeneity = 0.012). In the above-mentioned adjusted model, very similar results were obtained when HMW, rather than total, adiponectin was used as the exposure variable of interest.

**Conclusions:**

Our data suggest that the paradoxical association between high serum adiponectin levels and increased cardiovascular mortality rate is based on a cause-effect relationship, thus pointing to an unexpected deleterious role of adiponectin action/metabolism on atherosclerotic processes.

## Background

Despite insulin-sensitizing, anti-inflammatory and endothelial protective effects [1–4], adiponectin is an independent positive predictor of cardiovascular (CV) mortality in the general population [5] as well as several clinical sets [6–15] including type 2 diabetes (T2D) [16]. Because of such paradox, it is logical to suspect that high adiponectin rather than being a true risk factor is simply a marker of other metabolic disturbances, which, in fact, are able to causally shape the risk of CV mortality.

Genetic variants, which are robustly associated with a given modifiable variable, are strong and easy-to-use tools for assessing if causality underlines the association between that variable, used as an exposure, and outcomes of interest [17–19]. In our case, if genetic variants, firmly associated with circulating adiponectin levels, prove to be also associated with CV death, a strong case is made in favor of a causal role of adiponectin on CV mortality.

We and others have recently reported that single nucleotide polymorphism (SNP) rs822354, located in the locus of *ADIPOQ*, the gene which encodes for adiponectin [20], is associated with serum adiponectin at genome-wide level of statistical significance [21, 22].

Based on this background, we used rs822354 as an instrumental variable to investigate the causal nature of the counterintuitive independent association between adiponectin and CV mortality we have previously reported in patients with T2D [16].

## Methods

### Study population

#### Gargano Heart Study (GHS)-prospective design

This study comprises 368 patients with T2D (ADA 2003 criteria) and coronary artery disease (CAD) who were consecutively recruited at the Endocrine Unit of IRCCS “Casa Sollievo della Sofferenza” in San Giovanni Rotondo (Gargano, Center East Coast of Italy) from 2001 to 2008, as recently described [16, 23, 24]. All patients had either a stenosis >50 % in at least one coronary major vessel at coronary angiography or a previous myocardial infarction (MI). Follow-up information on outcomes was collected yearly from 2002 to 2011. The only exclusion criterion was the presence of poor life expectancy for non diabetes-related diseases. The end-point was CV mortality. Confirmation of the event was obtained from death certificates (i.e. according to the international classification of diseases’ codes: 428.1—ninth edition- and I21.0–21.9, I25.9, I46.9–50.9, I63.0, I63.9, I70.2-tenth edition). Clinical data at baseline were obtained from a standardized interview and examination. Smoking habits and history of hypertension, dyslipidemia and MI as well as glucose-lowering treatment were also recorded at time of examination. Data regarding medications were confirmed by review of medical records.

Serum total and high-molecular weight (HMW) adiponectin and genotyping of SNP rs822354 were assessed in 356 (96.7 %) participants.

The study was approved by the Institutional Ethic Committee IRCCS “Casa Sollievo della Sofferenza”, San Giovanni Rotondo.

#### Measurement of circulating total adiponectin levels

Serum total and HMW adiponectin concentrations were measured by ELISA (Alpco, Salem, NH) as previously described [25, 26]. The intra-assay coefficients of variation were 5.4 and 4.9, and 5.0 and 4.8 % for total adiponectin and HMW adiponectin, respectively [16].

#### Genotyping

SNP rs822354 was genotyped by Taqman SNP allelic discrimination technique by means of an ABI 7000 (Applied Biosystems, Foster City, CA). Call rate and concordance rate were ≥96 and >99 %, respectively. The SNP was in Hardy–Weinberg equilibrium (HWE) (p > 0.05).

#### Statistical methods

Patients’ baseline characteristics were reported as mean ± standard deviation (SD) and percentages for continuous and categorical variables, respectively. Deviation from HWE of the rs822354 was investigated by exact χ^2^ test.

Univariable linear regression analysis was performed to model the effect of the rs822354 polymorphism (assuming an additive genetic model of inheritance) on adiponectin levels (expressed in terms of log transformed because of its non-normal distribution) and results were reported as regression coefficient β (standard error, SE).

The time variable was defined as the time between the baseline examination and date of the event (CV mortality), or, for subjects who did not experience the event, the date of the last available clinical follow-up. Incidence rate (IR) for CV mortality was expressed as the number of events per total number of 100 person-years (py).

To estimate the causal effect of adiponectin levels on CV mortality, we performed mediation analyses using SNP rs822354 as instrumental variable.

Poisson regression models were performed to assess the association between rs822354 (per one risk allele increase), adiponectin values and the event occurrence. Risks were reported as incidence rate ratio (IRR) along with 95 % confidence interval (95 % CI).

Such models were used to estimate the effect size per minor allele of rs822354 polymorphism on adiponectin levels (β1) and on CV mortality (β2), and the effect size of adiponectin levels (rescaled by β1) on CV mortality (β3). Mendelian randomization holds if β2 and β3 are not statistically different. To test such difference, we introduced a bivariate generalized linear model with Poisson distribution, which allowed to simultaneously estimate both adjusted models (CV mortality vs. SNP rs822354 and CV mortality vs. adiponectin levels), using an appropriate statistical contrast.

Our sample size of 356 patients achieved a statistical power of 80 % (two-sided type I error rate = 0.05) to detect an allelic IRR on CV mortality at least equal to 1.70, given its annual IR of 3 % and a risk allele frequency of 0.37 we observed in our sample.

Two-sided p values <0.05 were considered for statistical significant.

All analyses were performed using SPSS version 15.0 (Chicago, IL) and SAS release 9.3 (SAS Institute, Cary, NC).

## Results

Clinical features of patients from GHS are summarized in Table 1. During follow-up (5.4 ± 2.5 years), 58 CV deaths occurred, corresponding to an overall annual IR of 3.0 % (58 events/1922 py).

**Table 1:** Baseline clinical characteristics of participants from GHS (N = 356)

Males (%)	67.4
Age (years)	64.5 ± 8.2
Smokers (%)	63 (17.7)
BMI (kg/m^2^)	30.2 ± 4.8
HbA_1C_ (%)	8.6 ± 1.9
Total cholesterol (mg/dl)	176.0 ± 45.8
HDL-cholesterol (mg/dl)	43.6 ± 14.6
Tryglicerides (mg/dl)	152.3 ± 91.8
Insulin w/wh oral agents (%)	192 (54.0)
Hypertension (%)	302 (84.8)
HMW-adiponectin (µg/ml)	3.1 ± 2.5
Total adiponectin (µg/ml)	4.9 ± 2.9

Continuous variables were reported as mean ± SD whereas categorical variables as total frequency and percentages

*GHS* Gargano heart study, *BMI* body mass index, *HbA1c* glycated haemoglobin A1c *HDL*-*cholesterol* high density lipoprotein-cholesterol, *HMW*-*adiponectin* high molecular weight-adiponectin

**Fig. 1:**
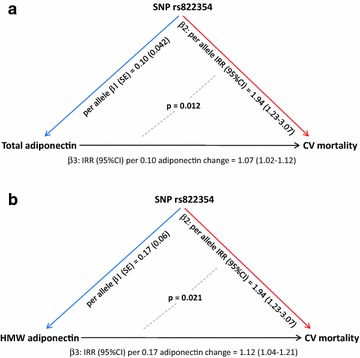
**a** Instrumental variable analysis for total adiponectin circulating levels. *β1* effect size of association between each minor allele of rs822354 and total adiponectin levels; *β2* effect size of association between each minor allele of SNP rs822354 and CV mortality; *β3* effect size of association between change of total adiponectin levels attributable to the effect of rs822354 (i.e. genetic equivalent of total adiponectin change) and CV mortality. **b** Instrumental variable analysis for HMW adiponectin circulating levels. *β1* effect size of association between each minor allele of rs822354 and HMW adiponectin levels; *β2* effect size of association between each minor allele of SNP rs822354 and CV mortality; *β3* effect size of association between change of HMW adiponectin levels attributable to the effect of rs822354 (i.e. genetic equivalent of HMW adiponectin change) and CV mortality

In these patients, the A allele of rs822354 was significantly associated with total adiponectin (β (SE) = 0.10 (0.042); p = 0.014). In addition, in a model comprising several variables relevant to the risk of death, including age, sex, smoking habits, BMI, HbA1c, total cholesterol, HDL-cholesterol, triglycerides, insulin therapy and hypertension, both the same allele at rs822354 (IRR = 1.94, 95 % CI 1.23–3.07; p = 0.005), as well as the genetic equivalent of total adiponectin change (IRR = 1.07, 95 % CI 1.02–1.12; p = 0.003), were significantly associated with CV mortality. Of note, the observed genetic effect was significantly greater than that exerted by the genetic equivalent change of serum adiponectin (i.e. change per one rs822354 allele) (p for IRR heterogeneity = 0.012) (Fig. 1a).

Similarly, rs822354 was associated with HMW adiponectin (β (SE) = 0.17 (0.06); p = 0.003), whose genetic equivalent change was, in turn, associated with CV mortality (adjusted IRR = 1.12, 95 % CI 1.04–1.21; p = 0.003). Also in this case, the association of CV mortality with either rs822354 or the genetic equivalent change of serum HMW adiponectin was significantly different (p for IRR heterogeneity = 0.021) (Fig. 1b).

## Discussion

Many cardiovascular risk factors recognize a genetic background [27–29]. Beside unraveling new pathogenic pathways, such genetic markers may be used as a tool for addressing the intrinsic nature underlying the relationship between metabolic variables and a given outcome of interest, an approach known as Mendelian randomization [30, 31].

Our data clearly show that the A allele of SNP rs822354 (in the *ADIPOQ* locus, which encodes for adiponectin [20]), is associated not only with high serum adiponectin levels (both total and HMW isoform), but also and most importantly with high CV mortality rate.

This finding is perfectly in line with a previous study showing that the G allele of SNP rs4783244 (in *CDH13*, encoding for the HMW adiponectin receptor T-cadherin [32]) is associated with both high HMW adiponectin levels [33–35] and increased mortality rate [35].

Although the exact mechanism through which SNP rs4783244 (or others in linkage disequilibrium, LD, with it) affects serum adiponectin is a matter of debate [33, 34], previous [35] and our present findings strongly suggest that the paradoxical association between increased mortality risk and high serum adiponectin, rather than recognizing the latter as only a marker of yet unknown biologically-unlinked abnormalities, is explained by a causal, deleterious effect of adiponectin action and/or metabolism [36, 37] on mortality rate.

Given the well-known beneficial effect of adiponectin on several metabolic and vascular phenotypes [4, 38–40], the intrinsic biological nature underlying such deleterious effect(s) is difficult to understand or even only to speculate; otherwise, we would not be talking of “paradoxical effect/association”. Having acknowledge this, some deleterious actions of adiponectin on processes possibly involved in atherogenesis, including stimulatory effect on secretion of pro-atherogenic cytokines [41–43] and angiogenesis [42, 44], have been recently reported in specific cell types. Whether such effects are observable also in human tissues involved in atherogenesis and whether they may play a role in increasing atherosclerotic processes is a possibility that deserves further, specifically designed studies, to be addressed. As a matter of fact, the available literature on the association with human traits does not unequivocally point to adiponectin as a metabolic and vascular protecting hormone. In fact, while low adiponectin has been associated with obesity, metabolic syndrome [39], some macro- and micro-vascular damages [38, 40] and T2D [39], so to make reasonable hypothesize that drugs activating adiponectin signaling pathways improve all the above mentioned metabolic and vascular disturbances [4], on the other hand, adiponectin levels are positively associated with subclinical atherosclerosis in patients with type 1 diabetes [45] and with all-cause mortality in patients with T2D [46, 47].

An additional, unexpected, finding of our study is that the association between SNP rs822354 and CV mortality is stronger than that expected if it would be entirely mediated by serum adiponectin changes. Such discrepancy might be due to the difference between a totally stable genetic effect, operating since birth on one hand and, conversely the role exerted by serum adiponectin, whose levels are quite variable [48, 49], especially in heavily treated patients as are those with T2D [50, 51], and whose effect has been evaluated only for a few years. As an alternative explanation, we cannot exclude that, beside the role on serum adiponectin, rs822354 exerts additional, distinct (i.e. pleiotropic) effects through other risk factors, which either we cannot account for or are still unknown. At this regard, it is of note that although neither rs822354, nor other SNPs in high LD with it, are associated with CV traits in humans [52, 53], it is located in the 3q27.3 region close to *KNG1*, a potential contributor in shaping CV risk [54]. In fact, *KNG1* encodes for high molecular weight kininogen, the precursor of bradykinin and kallidin, belonging to the pathway activating coagulation factors XI and XII [55] and interrelated with the renin–angiotensin–aldosterone system [56].

We like to acknowledge some limitations of our study. Firstly, the sample we analyzed comprises only 356 individuals, so that the strength of the novel association we here report (i.e. between rs822354 and CV mortality) does not reach genome-wide level of statistical significance. In addition, we lack a second, independent sample on which to replicate our finding. In all, although we cannot exclude the possibility of a false positive result, our study plays the important role of hypothesis generating, thus representing a solid background on which further similar studies can be based on.

Conversely, strengths of our study are the deep clinical phenotyping and the genetic and environmental homogeneity of our sample recruited in a well-defined geographical region. In addition, also the determination of both total and HMW adiponectin, which allowed us to confirm our finding also with the biological active isoform of adiponectin [57], reinforces our study.

Finally, the observation that among community-dwelling Japanese individuals [35], a genetic marker of adiponectin levels is associated with all-cause mortality, suggests that our present finding is generalizable across different ethnicities and is not restricted to CV mortality in the specific subset of patients with T2D.

## Conclusions

In conclusion, our present and previous [35] data suggest that adiponectin action/metabolism paradoxically causes increased CV mortality risk. Further studies are needed before this unexpected finding may be considered as established.

## Abbreviations

BMI: body mass index
CV: cardiovascular
GHS: Gargano heart study
HbA1c: glycated haemoglobin
HMW: high molecular weight
HWE: Hardy Weinberg equilibrium
IR: incidence rate
LD: linkage disequilibrium
MI: myocardial infarction
py: person-year
SNP: single-nucleotide polymorphism
T2D: type 2 diabetes
